# Intraperitoneal lactate/pyruvate ratio and the level of glucose and glycerol concentration differ between patients surgically treated for upper and lower perforations of the gastrointestinal tract: a pilot study

**DOI:** 10.1186/s13104-017-2622-9

**Published:** 2017-07-21

**Authors:** Jonas E. Sabroe, Anne R. Axelsen, Mark B. Ellebæk, Bjarne Dahler-Eriksen, Niels Qvist

**Affiliations:** 1 Department of Surgery, Odense University Hospital, 5000 Odense C, Denmark; 2 Department of Anaesthesiology and Intensive Care, Odense University Hospital, 5000 Odense C, Denmark

**Keywords:** Glycerol, Intraperitoneal microdialysis, Lactate/pyruvate ratio, Lactate/glucose ratio, Peritonitis

## Abstract

**Background:**

Secondary peritonitis is a condition associated with high morbidity and mortality. Continuous postoperative monitoring of patients to ensure timely intervention to treat complications without delay is important for survival and outcome. We aimed to (1) investigate potential differences in postoperative intraperitoneal biomarker levels between patients with upper and lower gastrointestinal tract lesion, and (2) compare postoperative biomarker levels between complicated and uncomplicated patients.

**Methods:**

We included a total of 15 consecutive patients operated for upper (n = 7) and lower (n = 8) gastrointestinal tract perforation. We registered postoperative complications during a 30 days follow up-period. Complications were defined as intraabdominal complications, septic shock, and mortality. 5 patients were complicated. A microdialysis catheter was placed intraperitoneally in each patient. Samples were collected every 4th hour for up to 7 postoperative days. Samples were analysed for concentrations of glucose, lactate, pyruvate and glycerol.

**Results:**

Microdialysis results showed that patients with upper gastrointestinal tract lesions had significantly higher levels of postoperative intraperitoneal glucose and glycerol concentrations, as well as lower lactate/pyruvate ratios and lactate/glucose ratios. In the group with perforation of the lower gastrointestinal tract, those patients with a complicated course showed lower levels of postoperative intraperitoneal glucose concentration and glycerol concentration and higher lactate/pyruvate ratios and lactate/glucose ratios than those patients with an uncomplicated course.

**Conclusion:**

Patients with upper and lower gastrointestinal tract lesions showed differences in postoperative biomarker levels. A difference was also seen between patients with complicated and uncomplicated postoperative courses.

## Background

Secondary peritonitis (SP), the most commonly encountered type of peritonitis [1], is defined as an inflammation of the peritoneum due to an intraabdominal pathological condition [1–3]. The underlying condition in SP is most often a rupture of a hollow organ either spontaneously or following surgery or trauma [2, 4], and perforation of the large bowel is the most frequent [5, 6].

SP is associated with high mortality and morbidity. In-hospital mortality varies between 15% and 47.4% depending on the patient material [3, 5, 7, 8]. Morbidity includes renal failure, cardiovascular failure, intraabdominal abscesses, and sepsis [4–7].

The primary treatment is surgical source control. Continuous postoperative monitoring of patients to ensure timely intervention to treat complications without delay is important for survival and outcome [9]. Intraperitoneal microdialysis (IPM) makes it is possible to obtain continuous monitoring of the intraabdominal condition by measuring several parameters as markers for inflammation or ischaemia. Studies in humans have shown promising results of IPM compared to conventional postoperative monitoring with clinical observations and paraclinical examinations in early diagnosis of anastomotic leakage [10–13].

To our knowledge no previous published study has evaluated IPM as a clinical tool for postoperative monitoring of patients treated for secondary or tertiary peritonitis. The present study aimed to assess microdialysis in this setting. This pilot study was conducted in order to see, if the study could be carried out in a practical and safe manner. We also aimed to evaluate the results from IPM with continuous measurement of lactate, pyruvate, glucose and glycerol concentration in the peritoneal fluid in patients operated for peritonitis due to gastrointestinal tract (GI) perforation. The present study should provide data and information useful in the planning of future studies including power calculations. The primary aim was to investigate potential differences between patients with lower GI lesions (distal to the ligament of Treitz) and those with upper GI lesions. We hypothesized that a difference between these two groups of patients exists. If this is true, one should interpret microdialysis results according to the location of the perforation. A secondary aim was to determine potential differences in postoperative intraperitoneal biomarker levels between complicated and uncomplicated patients.

## Methods

### The microdialysis principle

Microdialysis is a minimally invasive technique that allows for in vivo sampling of unbound compounds from the interstitial space. The microdialysis system includes a double-lumen microdialysis catheter, a syringe pump, and microvials for collection of dialysates. The catheter is placed in the tissue or cavity of interest, and the syringe pump ensures a constant flow of perfusion fluid in the catheter. At the tip of the catheter, molecules diffuse across a semipermeable membrane from the interstitial space to the perfusion fluid inside the catheter. The transfer of molecules is passive from high to low concentrations. In this way, the microdialysis catheter mimics a capillary. The perfusion fluid is collected in microvials and analysed bedside, thus providing a dynamic view of changes in concentrations of molecular substances within the interstitial spaces. It has proven to be a safe procedure with a low rate of minor complications and no major complications [11, 14].

### Biomarkers

Traditionally, glycerol, lactate, pyruvate and glucose are the substances that most frequently have been measured using the microdialysis principle [10, 13, 15–23] as commercially available equipment provides bedside measurements of these substances. Under anaerobic conditions (e.g. due to compromised perfusion of tissue) pyruvate is converted to lactate. In cases of low levels of oxygen or insufficient energy supply, high levels of lactate and low levels of glucose and pyruvate are observed. These changes may be an early indication of postoperative complications. When the body is in a catabolic state (e.g. following surgery), cleavage of triglyceride results in the release of glycerol [13, 24–26]. Glycerol may also derive from the breakdown of phospholipids (the major component of cell walls) when the cell is depleted of glucose or oxygen [20]. Therefore, glycerol should increase in patients with postoperative complications; however, the opposite has also been demonstrated [11, 13], and the mechanism behind the increase and decrease in intraperitoneal glycerol concentration following surgery is not fully understood.

### Setting

This prospective observational single-centre study was conducted at the surgical department of Odense University Hospital. The study was conducted according to the Declaration of Helsinki following approvals from the Danish Health and Medicines Authority (EudraCT No.: 2012-004398-22), the Regional Scientific Ethical Committees for Southern Denmark (ID: S-20130018), and the Danish Data Protection Agency (2008-58-0035).

### Patients and procedures

Inclusion criteria included: more than 18 years of age; informed written consent obtained from the patient or relatives; contamination of two of the four abdominal quadrants with overt peritonitis. Exclusion criteria included known severe renal disease (estimated glomerular filtration rate (eGFR) < 30 mL/min/1.73 m^2^) and known intolerance to standard antibiotic regimens.

**Fig. 1:**
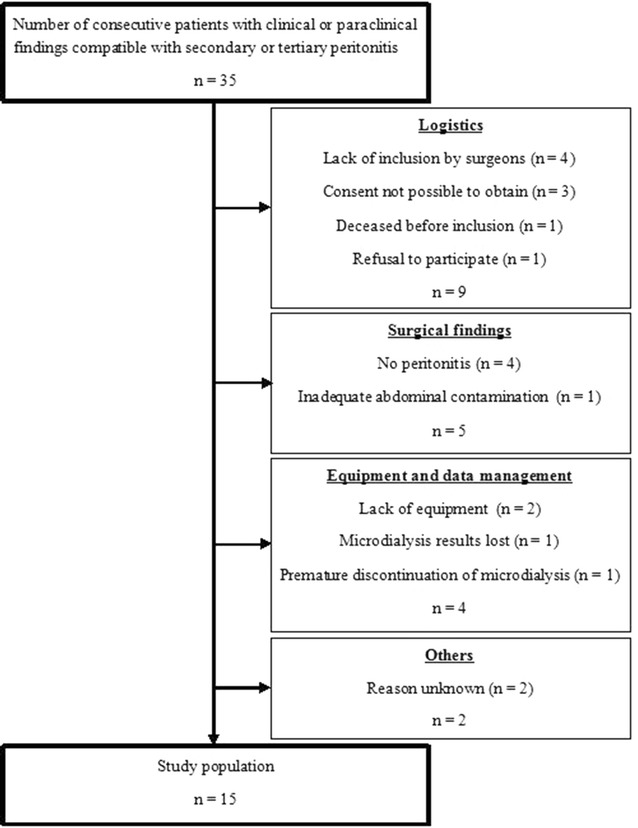
Flowchart of participant inclusion

From July 15th 2013 to April 14th 2014, we registered 35 consecutive patients eligible for the study (Fig. 1). From this cohort we excluded 20 patients. 1 patient died prior to inclusion. 4 patients did not have peritonitis, and 1 patient had local peritonitis confined to one abdominal quadrant only. 3 were unable to consent and 1 patient refused to participate. In 2 cases, we lacked equipment. The attending surgeons did not include 4 patients because of various logistic reasons. Microdialysis data was lost from 1 patient, and 1 patient had accidental preterm removal of the microdialysis catheter. 2 patients were not included for reasons unknown. In total, we included 15 evaluable patients.

Comorbidities, including cardiovascular disease (CVD), chronic obstructive pulmonary disease (COPD), diabetes and active cancer, were registered. CVD was defined as hypertension, angina, arrhythmia, claudication, previous myocardial infarction, previous apoplexy, previous transitory cerebral ischaemia, previous cardiac bypass operation and/or previous coronary angiography.

It was left to the surgeon’s discretion to choose between laparoscopy and laparotomy. All patients received intravenous antibiotic prophylaxis with 3 g cefuroxim (Zinacef^®^, Actavis, Gentofte, Denmark) and 1.5 g metronidazole (Baxter A/S, Allerød, Denmark) either within 30 min prior to skin incision or when peritonitis was diagnosed during surgery. At the end of the surgical procedure, a microdialysis catheter was placed into the peritoneal cavity by use of a splittable introducer (Flocare Jejunokath^®^, Nutricia, Erlangen, Germany). The tip of the catheter was placed free floating in the most contaminated region of the abdomen. The microdialysis catheter was fixated to the skin to avoid dislocation. Every patient had a central venous line (CVL) inserted. Following surgery, patients were either transferred to an intensive care unit (ICU) or recovery ward depending on the clinical status of the individual patient.

### Microdialysis set-up

A microdialysis catheter (CMA 65 custom-made microdialysis catheter, M Dialysis AB, Stockholm, Sweden) with a cut-off value of 100 kDa, a membrane length of 30 mm (material: polyethersulfone), and a shaft length of 310 mm was used in this study. The catheter was perfused continuously with Voluven^®^ (Fresenius Kabi, Island Brygge, Denmark) via a pump (CMA 106 or 107 microdialysis syringe pump, CMA Microdialysis AB, Stockholm, Sweden) with a flow rate of 0.3 µL/min. Dialysates were analysed every 4th hour at the bedside for the concentrations of lactate, pyruvate, glucose and glycerol using an ISCUS^flex^ microdialysis analyser (M Dialysis AB, Stockholm, Sweden). The lactate/pyruvate (L/P) ratio and lactate/glucose (L/G) ratio were calculated. The microdialysis continued for a maximum of 7 days.

### Data collection and management

The American Society of Anesthesiologists (ASA) score [27], acute-phase reactants [C-reactive protein (CRP), white blood cell (WBC) count], acid–base status, and eGFR were obtained prior to surgery. Sequential Organ Failure Assessment (SOFA) score was registered on postoperative day (POD) 1 [28]. We registered clinical events during a 30-day follow-up period (complications related to the surgical intervention, re-leakage from the GI tract or formation of intraabdominal fistula, intraabdominal abscess or empyema, intestinal ischemia, re-operation, mortality, and septic shock). Septic shock was defined as sepsis and continues hypotension (systolic blood pressure <90 mmHg or mean arterial pressure <70 mmHg or decrease in systolic blood pressure >40 mmHg or lactate concentration >4 mmol/L in phripheral blood) despite of fluid resuscitation or need for inotropic and/or pressor agents. Analyses of the dialysate from the microdialysis catheter were compared to the clinical course.

### Statistics

Continuous variables are presented as medians with interquartile range (IQR 25th, 75th) or range. Medians were compared using the Mann–Whitney U test. Data analysis was performed using IBM SPSS Statistics 21. A two-sided *P* value of less than 0.05 was considered statistically significant. We present data from POD 1–5 as all patients underwent at least 5 days of IPM.

## Results

Three patients underwent primary laparoscopy (No. 1, 2 and 4), and two were converted to open surgery (No. 1 and 4). The rest of the patients (n = 12) underwent primary laparotomy. Eight patients and seven patients had an organ lesion distal (lower) and proximal (upper) to the ligament of Treitz, respectively. The group of patients with lower GI perforation included three males and five females, median age was 60 years (range 49–80), and median body mass index (BMI) was 24.7 (range 22.6–27.5). The group of patients with upper GI perforation included two males and five females, median age was 63.5 years (range 50–79), and median BMI was 22.7 (range 15.2–27.7). The median microdialysis sampling period was 7 days (range 5–7). The median number of postoperative days in the ICU was 0 (range 0–7). Baseline patient characteristics are presented in Table 1. Seven patients were current smokers. Four patients had active cancer at inclusion. Eight patients had CVD. One patient had diabetes, and one had COPD. Table 2 summarises the clinical and paraclinical findings at inclusion. The median ASA score was 3.5 and 3 for patients with upper and lower GI lesion, respectively. The median SOFA score on POD 1 was 4.5 and 9 for patients with upper and lower GI lesion, respectively. Details regarding surgical procedures are provided in Table 3.

**Table 1:** Baseline characteristics

	Smoking status	Ongoing cancer	CVD	Diabetes	COPD
Upper perforation					
Patient 1	Yes	–	Yes	–	–
Patient 2	Yes	–	Yes	–	–
Patient 3	–	–	Yes	–	–
Patient 4	Yes	–	–	–	–
Patient 5	–	–	Yes	–	–
Patient 6	Yes	–	–	–	–
Patient 7	–	–	–	–	–
Lower perforation					
Patient 8	Yes	–	Yes	–	–
Patient 9	–	Yes	–	–	–
Patient 10	Yes	–	Yes	–	–
Patient 11	–	Yes	Yes	–	–
Patient 12	Yes	Yes	–	Yes	–
Patient 13	–	–	–	–	–
Patient 14	Yes	–	Yes	–	Yes
Patient 15	–	Yes	–	–	–

*CVD* cardiovascular disease, *COPD* chronic obstructive pulmonary disease

**Table 2:** Clinical and paraclinical findings at inclusion

	MAP (mmHg)	ASA score	pH	Lactate (mmol/L)	CRP (mg/L)	WBC count (10E9/L)	eGRF (mL/min/1.73 m^2^)	HCO_3_^−^ (mmol/L)	SOFA score POD 1
Upper perforation									
Patient 1	105.7	3	7.44	2.4	37	18.5	82	25.1	n/a
Patient 2	87.0	n/a	7.39	3.4	<1	12.8	91	16.2	2
Patient 3	64.3	4	n/a	2.4	115	6.1	60	20.2	6
Patient 4	68.0	n/a	n/a	n/a	138	1.2	55	n/a	1
Patient 5	124	3	7.50	1.3	<1	7.9	58	25.0	1
Patient 6	105	2	7.41	0.8	2.2	18.7	95	20.4	2
Patient 7	66.7	4	7.28	1.9	246	17.5	17	17.7	7
Median	87.0	3	7.41	2.15	37	12.8	60	20.3	2
Lower perforation									
Patient 8	56.0	3	7.34	5.2	295	6.4	41	n/a	15
Patient 9	87.0	3	n/a	n/a	262	5.5	38	n/a	2
Patient 10	n/a	4	7.45	3.4	1.9	16.6	116	21.8	5
Patient 11	136.7	2	7.37	2.5	3.5	8.0	71	23.5	8
Patient 12	102.3	2	7.39	1.7	110	9.4	104	20.1	2
Patient 13	112.3	2	n/a	n/a	30	10.1	66	n/a	3
Patient 14	49.7	3	7.37	1.3	598	6.2	33	n/a	5
Patient 15	86.0	3	n/a	n/a	202	7.7	71	n/a	3
Median	87.0	3	7.37	2.5	156	7.85	68.5	21.8	4

*MAP* mean arterial pressure, *ASA* American Society of Anesthesiologists, *CRP* C-reactive protein, *WBC* white blood cell, *eGFR* estimated glomerular filtration rate, *n/a* data not available, *SOFA* sequential organ failure assessment, *POD 1* postoperative day 1

**Table 3:** Surgical findings and interventions

	Cause of perforation	Exploration method	Surgical intervention	Type of closing	Duration of surgery	Irrigation	Drainage
Upper perforation							
Patient 1	Ulcer, prepyloric	Diag. lap. converted to expl. lap.	Suture of ulcer	PDS + staples	1 h 20 min	Yes	–
Patient 2	Ulcer, duodenal bulb	Diag. lap.	Suture of ulcer	Vicryl	1 h 10 min	Yes	–
Patient 3	Ulcer, duodenum	Expl. lap.	Billroth II resection	PDS	1 h 51 min	Yes	Yes
Patient 4	Ulcer, prepyloric	Diag. lap. converted to expl. lap.	Suture of ulcer	PDS	1 h 20 min	Yes	–
Patient 5	Ulcer, prepyloric	Expl. lap.	Suture of ulcer	PDS	45 min	Yes	–
Patient 6	Ulcer, prepyloric	Expl. lap.	Suture of ulcer	PDS	1 h 3 min	Yes	–
Patient 7	Ulcer, duodenal bulb	Expl. lap.	Suture of ulcer	PDS	n/a	Yes	–
Lower perforation							
Patient 8	Rectum perforation do to coprostasis	Expl. lap.	Hartmann’s procedure	PDS + staples	2 h 56 min	Yes	–
Patient 9	Perforated c. recti	Expl. lap.	Hartmann’s procedure	PDS	2 h 40 min	Yes	–
Patient 10	Perforated diverticulitis	Expl. lap	Hartmann’s procedure	PDS	n/a	Yes	–
Patient 11	Perforated c. recti	Expl. lap.	Hartmann’s procedure	VAC	2 h 40 min	Yes	–
Patient 12	Rectal stump blow out	Expl. lap.		PDS	n/a	Yes	–
Patient 13	Perforated diverticulitis	Expl. lap.	Hartmann’s procedure	PDS	n/a	Yes	–
Patient 14	Perforated coecum do to ischemia	Exp.lap.	Right sided hemicolectomy withileostomy	VAC	2 h 5 min	Yes	–
Patient 15	Perforated diverticulitis	Exp. Lap.	Hartmann’s procedure	PDS	3 h 9 min	Yes	–

*Diag. lap.* diagnostic laparoscopy, *Expl. lap.* explorative laparotomy, *PDS* polydioxanone, *VAC* vacuum assisted closure

Five patients (No. 8, 10, 11, 12 and 14) experienced postoperative complications during a 30-day follow-up period. All complicated patients had lower GI perforation. Three patients (No. 8, 10 and 11) developed one or more intraabdominal abscesses. One patient (No. 11) was re-operated due to a bladder lesion during primary surgery. One patient (No. 12) developed a fistula between the bladder and the rectum. One patient (No. 14) died due to sepsis and multiple organ failure.

A possible IPM-related complication occurred in one patient (No. 8), who developed an intraabdominal empyema around the microdialysis catheter.

**Fig. 2:**
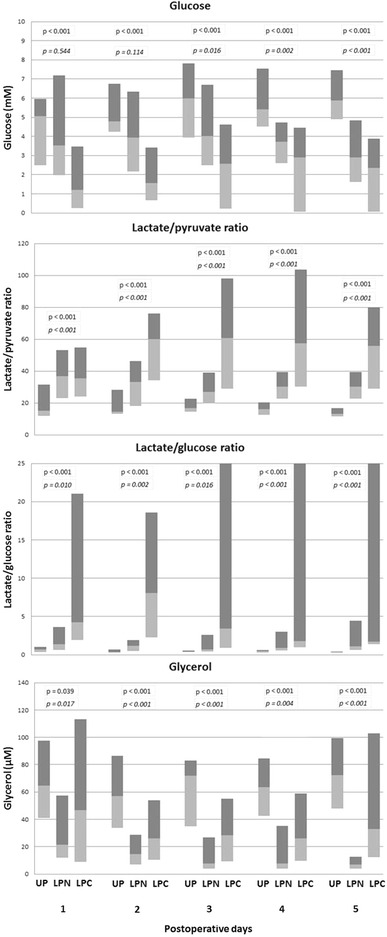
Microdialysis results. Postoperative intraperitoneal glucose concentration, lactate/pyruvate ratio, lactate/glucose ratio and glycerol concentration for patients with upper and lower gastrointestinal tract lesions (medians, upper and lower quartiles). Patients with a lower perforation are stratified into groups with and without complications. The P values in italics refer to the comparison of patients with an upper perforation to patients with a lower perforation and no postoperative complications. The other P values refer to the comparison of patients with an upper perforation to patients with a lower perforation and with postoperative complications. *UP* upper perforation postoperative, *LPN* lower perforation and no complications, *LPC* lower perforation and with complications

Patients with upper GI perforation showed significantly higher levels of postoperative intraperitoneal glucose concentration from POD 1 and 3 compared to patients with lower GI perforation with and without postoperative complications, respectively (Fig. 2). The group of patients with upper GI perforation showed significantly lower levels of postoperative intraperitoneal L/P ratio compared to both groups of patients with lower GI perforation (Fig. 2). The same applied for the postoperative intraperitoneal L/G ratio, although no statistically significant difference was found on POD 1 for patients with upper GI perforation compared to uncomplicated patients with lower GI perforation (Fig. 2). Patients with upper GI perforation showed significantly higher levels of postoperative intraperitoneal glycerol concentration compared to both groups of patients with lower GI perforation (Fig. 2).

Reliable statistical analysis of potential differences in the postoperative intraperitoneal biomarker levels between complicated and uncomplicated patients with lower GI perforation was not possible due to the low number of patients. However, we observed a trend that patients with complications had lower levels of glucose concentration and higher levels of glycerol concentration, L/P ratio and L/G ratio (Fig. 2).

## Discussion

Postoperative IPM showed significant differences between patients with upper GI perforation compared to patients with lower GI perforation. Moreover, we registered a trend in the difference between complicated and uncomplicated patients with lower GI perforation. In general, patients with lower GI perforation had lower levels of postoperative intraperitoneal glycerol and glucose concentration, as well as higher L/P and L/G ratios. Uncomplicated patients with lower GI perforation presented higher concentrations of postoperative glucose and lower glycerol concentrations, L/P ratios and L/G ratios compared to complicated patients with lower GI perforation, and this could be a result of either ischaemia or inflammation, or a combination of both.

It has previously been shown that patients who develop pancreatic fistula following Whipple’s procedure have a high concentration of postoperative intraperitoneal glycerol [29], which may be due to leakage of pancreatic enzymes into the abdominal cavity. This could also be the explanation for higher levels of glycerol concentration seen in patients with upper GI perforation compared with patients with lower GI perforation. We believe that results from the present study indicate that IPM has the potential to become a supplement to physical examination and conventional paraclinical tests in monitoring of patients treated for SP. However future studies are warranted. The results of this study indicates, that future studies should distinguish between patients with upper and lower GI perforation, and microdialysis measurements from these two groups of patients should be interpreted separately.

To our knowledge, only two studies on IPM after urgent surgery for intraabdominal conditions have been published. Verdant et al. [14] conducted a study in patients undergoing acute laparotomy, and the study population included a mixture of patients with bowel perforation, primary peritonitis, mesenteric ischaemia, haemorrhage, pancreatitis, cholecystitis, bowel obstruction, and complications to caustic ingestion. In those patients who developed complications, a mean L/P ratio of 35 was found at POD 1, increasing to approximately 50 at POD 5. In patients with an uncomplicated course, the mean L/P ratio was 18 at POD 1 and remained at a steady level for the rest of the study period. In the present study, patients with a complicated course (all diagnosed with lower GI perforation) had slightly higher intraperitoneal L/P ratios on POD 1 to POD 5 compared with the complicated patients in the study of Verdant et al. An explanation for this may be that not all patients with a complicated course in the study of Verdant et al. presented with contamination of the abdominal cavity prior to surgery. Patients with upper GI perforation from the present study (all uncomplicated) had lower postoperative L/P ratios than uncomplicated patients in the study of Verdant et al. Conversely, uncomplicated patients with lower GI perforation from the present study had higher postoperative L/P ratios compared with uncomplicated patients in the study of Verdant et al. This could indicate that lower GI perforation causes a more severe inflammatory reaction and/or more ischaemia within the abdominal cavity compared with upper GI perforation.

In a study by Konstantinos et al. [30], a microdialysis catheter was inserted into the abdominal cavity in patients admitted to the ICU with an underlying intraabdominal condition. Twenty-one patients were included in the study, of which 13 patients underwent surgery. Nine patients died. Underlying pathological conditions included colectomy, acute pancreatitis, gastric haemorrhage, acute abdomen, ileus, and multi-trauma. Levels of intraperitoneal glucose, glycerol, lactate and pyruvate were observed. For each patient, the authors calculated the mean L/P ratio during the first 3 days following admission to the ICU. The authors proposed a postoperative cut-off value of 25.94 for the risk of death. Both complicated and uncomplicated patients with lower GI perforation in the present study presented with postoperative median L/P ratios on POD 1–5 that exceeded the proposed cut-off value of Konstantinos et al. However, only one patient died in the present study. The high postoperative intraperitoneal L/P ratio registered in patients with lower GI perforation in the present study is likely to be caused by the faecal abdominal contamination of these patients.

The study population of the present study was more homogeneous than the study population of the two referred studies [14, 30]. It should be expected that different abdominal pathological conditions lead to different results of IPM. We therefore suggest that future studies in IPM stratify patients according to underlying abdominal pathological condition.

Eight patients were not included in the study due to lack of equipment, no inclusion of attending surgeons, loss of microdialysis data, and preterm removal of the microdialysis catheter. Furthermore 2 patients were not included for reasons unknown and 3 patients were unable to consent. This shows that use of microdialysis in studies of patients undergoing acute surgery is difficult. Particularly recruitment of patients is challenging.

### Strengths and limitations

The major strength of the present study is that patients were included consecutively and stratified to upper and lower GI perforation. Nevertheless, some significant limitations exist. Inclusion of patients undergoing acute surgery for SP is challenging. Obtaining informed consent is difficult due to the clinical condition of the patient, which may exclude the sickest patients. Only one patient died during the postoperative course, which indicates a certain selection among the study population. Finally, the sample size of the study is small and heterogeneous with regard to baseline characteristics. Due to the small sample size we did not perform statistical analysis with stratification of patients according to baseline characteristics. So far no definitive conclusion should be made, but the results encourage further studies on the applicability of IPM in monitoring of patients surgically treated for SP.

## Conclusion

Patients with upper and lower GI perforation showed differences in the results of IPM during the postoperative period. IPM results were also found to differ between complicated and uncomplicated patients with lower GI perforation. Future studies on IPM are warranted to define the clinical implications.

## Abbreviations

ASA: American Society of Anesthesiologists
BMI: body mass index
CRP: C-reactive protein
CVD: cardiovascular disease
CVL: central venous line
COPD: chronic obstructive pulmonary disease
eGFR: estimated glomerular filtration rate
GI: gastrointestinal tract
ICU: intensive care unit
IPM: intraperitoneal microdialysis
IQR: interquartile range
L/G: lactate/glucose
L/P: lactate/pyruvate
POD: postoperative day
SP: secondary peritonitis
WBC: white blood cell
